# Supplementary material for: “Candidatus Dechloromonas phosphoritropha” and “Ca. D. phosphorivorans”, novel polyphosphate accumulating organisms abundant in wastewater treatment systems
Source: ISME J. 2021 Jun 21;15(12):3605–14. doi: 10.1038/s41396-021-01029-2 (PMC8630035; doi:10.1038/s41396-021-01029-2)
Supplement: Supplementary file 3 — Supplementary material [file 41396_2021_1029_MOESM3_ESM.pdf]

## SUPPLEMENTARY MATERIAL FOR

# "*Candidatus* Dechloromonas phosphoritropha" and "*Ca.* D. phosphorivorans", novel polyphosphate accumulating organisms abundant in wastewater treatment systems


Francesca Petriglieri, Caitlin Singleton, Miriam Peces, Jette F. Petersen, Marta Nierychlo, Per H. Nielsen.

Center for Microbial Communities, Department of Chemistry and Bioscience, Aalborg University, Aalborg, Denmark.


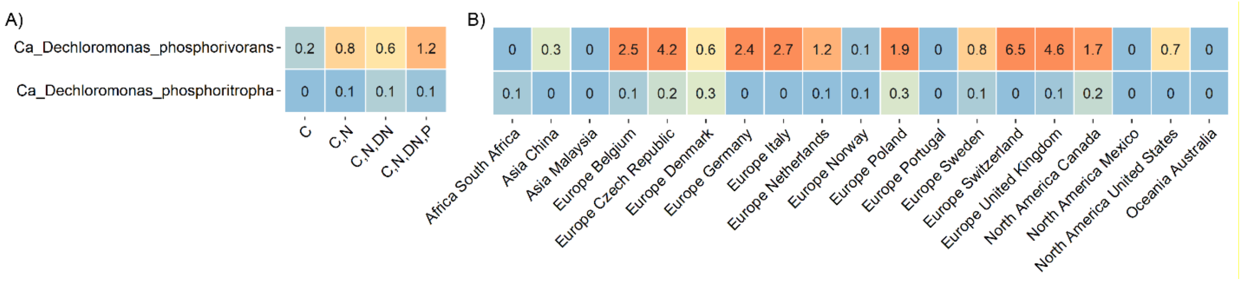


**SFigure 1.** Average relative read abundance of *Ca.* Dechloromonas phosphorivorans and *Ca.* Dechloromonas phosphoritropha across A) different process configurations (N plants = 480; C - carbon removal; C,N - carbon removal and nitrification; C,N,DN - carbon and nitrogen removal; C,N, DN - carbon, nitrogen and phosphorus removal), and B) across the world in EBPR (C,N,DN,P) plants (N plants = 111). Data comes from the global MiDAS survey of microbial communities in WWTPs (Dueholm et al., 2021).

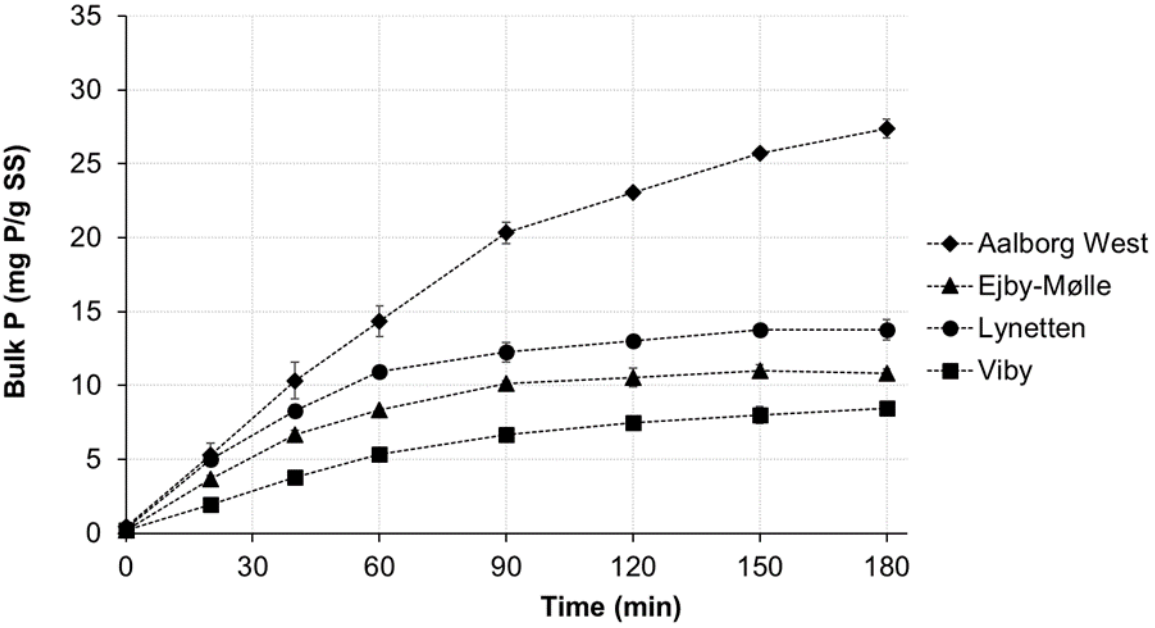


**SFigure 2**. Anaerobic phosphate release profiles from 4 full-scale EBPR plant activated sludge samples, amended with a mixture of acetate, glucose, and casamino acids with a final concentration of 500, 250, and 250 mg $L^{-1}$, respectively.

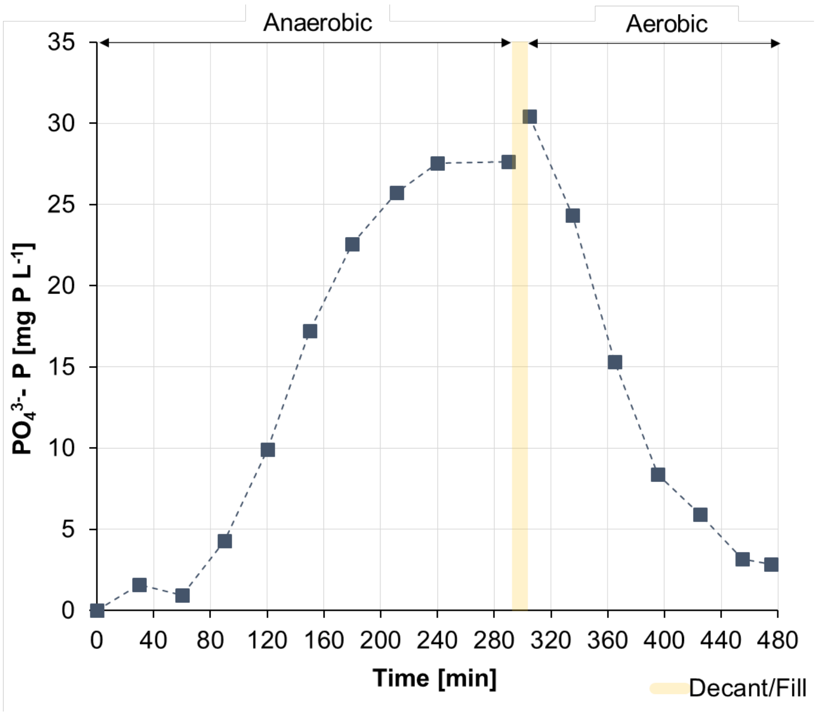


**SFigure 3**. Phosphate release and phosphate uptake from SBR biomass containing PAOs during an operational cycle. The substrate added under anaerobic conditions was casein hydrolysate at an initial concentration of 200 mg COD $L^{-1}$

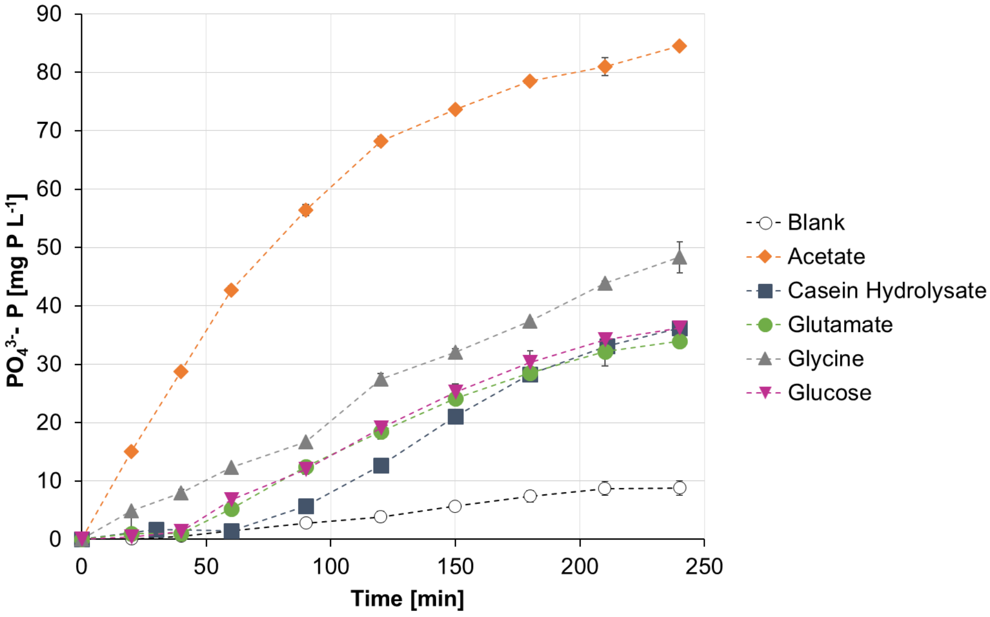


**SFigure 4**. Anaerobic phosphate release profiles obtained with different carbon sources with an initial cycle concentration of 200 mg COD $L^{-1}$ from SBR biomass containing PAOs, including *Dechloromonas*.

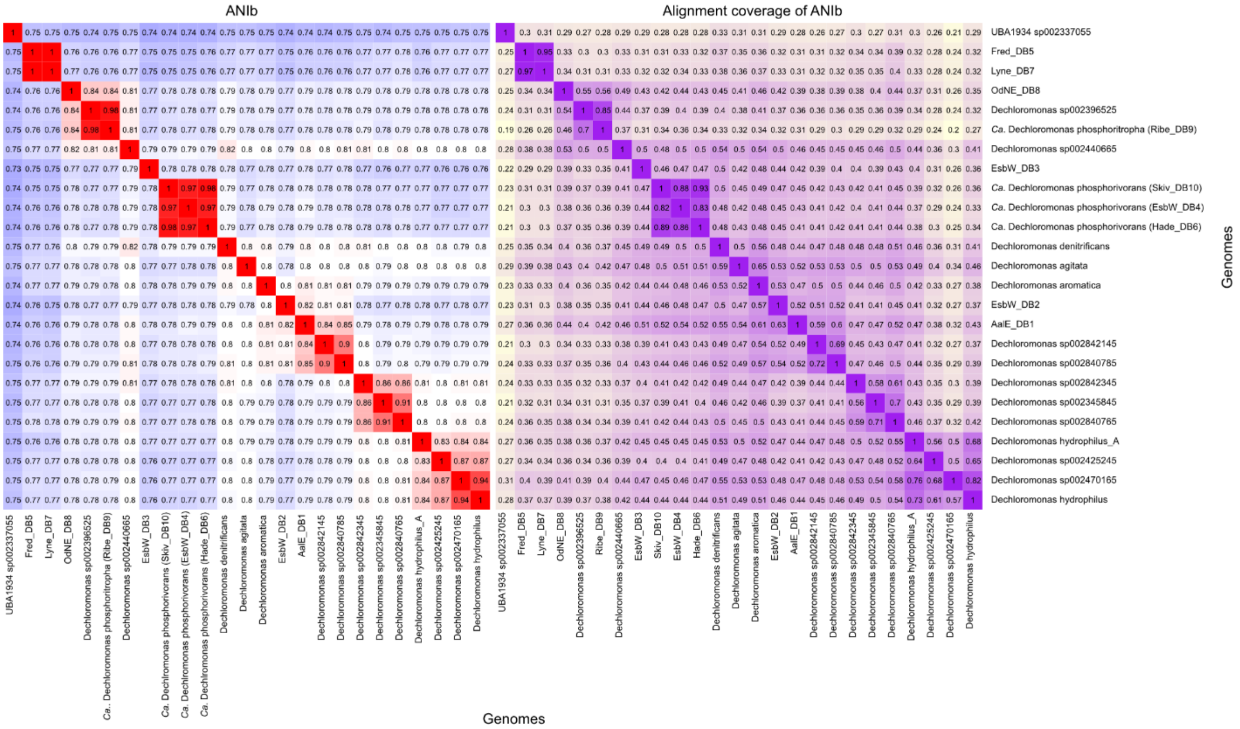


**SFigure 5**. Average nucleotide identity (%) using BLASTN+ (ANIb) and alignment coverage (%) of the genomes and MAGs in Figure 5. The genomes are ordered by the genome tree in Figure 5.

**SDataFile 1**. 100% complete KO modules identified using EnrichM

**SDataFile 2**. 16S rRNA genes used for record of provisional taxon for *Ca.* Dechloromonas phosphoritropha and *Ca.* Dechloromonas phosphorivorans.

**STable 1.** KO annotations for the MAGs and gene acronyms used throughout the manuscript.

**STable 2**. Complete protologues for inclusion in the codified record of provisional taxon for *Candidatus* Dechloromonas phosphoritropha and *Candidatus* Dechloromonas phosphorivorans.

| | | |
|---|---|---|
| **Taxonumber** | N/A | N/A |
| **Species name** | *Candidatus* Dechloromonas phosphoritropha | *Candidatus* Dechloromonas phosphorivorans |
| **Genus name** | *Dechloromonas* | *Dechloromonas* |
| **Specific epithet** | phosphoritropha | phosphorivorans |
| **Type species of the genus** | *Dechloromonas agitata* strain CKB | *Dechloromonas agitata* strain CKB |
| **Taxonumber of the type species** | 73030 | 73030 |
| **Genus status** | Validly published | Validly published |
| **Species etymology** | phos.pho.ri,tro'pha. from N. L. masc. N. *phosphorus*, phosphorus; Gr. Masc. n. *trophos*, one who feeds; N. L. fem. adj. *phosphoritropha*, indicating the ability to accumulate poly-phosphate | phos.pho.ri.vo´.rans, from N.L. masc. n. *phosphorus*, phosphorus; L. pres. part. *vorans,* devouring; N.L. part adj. *phosphorivorans*, indicating the ability to accumulate poly-phosphate |
| **Species status** | sp. nov. | sp. nov. |
| **Designation of the type MAG** | GCA_016722705.1 | GCA_016721185.1 |
| **MAG/SAG accession number** | GCA_016722705.1 | GCA_016721185.1 |
| **Genome status** | High-quality draft | High-quality draft |
| **Genome size** | 4,726,453 | 4,220,355 |
| **GC mol %** | 61.9 | 60.8 |
| **Country of origin** | Denmark | Denmark |
| **Region of origin** | Ribe | Skive |
| **Source of sample** | Full-scale biological nutrient removal wastewater treatment plant | Full-scale biological nutrient removal wastewater treatment plant |
| **Geographical location** | Ribe | Skive |
| **Latitude** | 55.3269° N | 56.5651° N |
| **Longitude** | 8.7747° E | 9.0309° E |
| **Depth** | N/A | N/A |
| **Altitude** | N/A | N/A |
| **Temperature of the sample [In celcius degrees]** | Mesophilic | Mesophilic |
| **pH of the sample** | N/A | N/A |
| **Relationship to oxygen** | Facultative anaerobe | Facultative anaerobe |
| **Energy metabolism** | Potentially utilizing acetate and amino acids | Potentially utilizing acetate and amino acids |
| **Assembly** | 1 sample | 1 sample |
| **Sequencing technology** | Oxford Nanopore and Illumina Hiseq X | Oxford Nanopore and Illumina Hiseq X |
| **Binning software used** | Maxbin v2.2.7 | Maxbin v2.2.7 |
| **Assembly software used** | CANU v1.8 | CANU v1.8 |
| **Habitat** | Full-scale biological nutrient removal wastewater treatment plant | Full-scale biological nutrient removal wastewater treatment plant |
| **Miscellaneous, extraordinary features relevant for the description** | Rod-shaped often arranged in spherical microcolonies | Rod-shaped often arranged in spherical microcolonies |

## References

Dueholm, M.S., Nierychlo, M., Andersen, K.S., Rudkjøbing, V., Knudsen, S., the MiDAS Global Consortium, Albertsen, M., Nielsen, P. H. (2021): MiDAS 4 - a global WWTP ecosystem-specific full-length 16S rRNA gene catalogue and taxonomy for studies of bacterial communities. *BioRxiv.*
